# Key drivers of brand trust in a Latin American airline: the impact of Colombia’s Avianca customer experience

**DOI:** 10.1057/s41270-023-00208-8

**Published:** 2023-02-18

**Authors:** Jose Ribamar Siqueira, Michael Bendixen, Felipe Reinoso-Carvalho, Raffaele Campo

**Affiliations:** 1 Departament of Business Administration, Pontificia Universidad Javeriana, Carrera 7 No. 40 – 62, Bogotá, Colombia; 2 Gordon Institute of Business Science, University of Pretoria, Johannesburg, South Africa; 3 School of Management, University of los Andes, Bogotá, Colombia; 4 Department of Economics and Finance, University of Bari, Largo Abbazia Santa Scolastica 53, Bari, Italy

**Keywords:** Airline industry, Service quality, Customer experience, Other customers, Brand trust

## Abstract

Trust in a company's brand is essential for businesses that rely on repeat business from customers. In light of this, this study aims to investigate the key factors that drive customer trust in airline brands within the Latin American context. In order to accomplish this goal, an augmented version of AIRQUAL was utilized to evaluate Colombian native customers' perceptions of the customer experience (CX) provided by Avianca, a well-known and highly regarded airline active in Latin America. AIRQUAL is a model used to evaluate the quality of airline service proposed by Nadiri et al. (2008). It consists of five dimensions: airline tangibles, terminal tangibles, personnel services, empathy, and image. These dimensions were expanded to capture additional touchpoints identified in the literature. They represent internal and external touchpoints that make up the airline customer experience, resulting in a more robust research model. The additional dimensions, namely the impact of perceptions associated with other customers and the process of the purchase experience, were incorporated to account for a more holistic assessment of the experience provided. They also help capture the three stages of the experience provided by the airline as proposed by Namukasa (2013) before, during, and after the flight. The examined drivers of brand trust in the proposed model were the dimensions of the augmented AIRQUAL model and a measure of CX. Results indicate that while most dimensions impact brand trust, CX was identified as the key driver of brand trust and acted as a mediator of the dimensions of the augmented AIRQUAL and brand trust. According to the findings of this research, all three aspects of service quality—pre-flight service quality, in-flight service quality, and post-flight service quality—are of comparable significance and have a significant bearing on how customers evaluate their experiences.

## Introduction

The airline industry is presently facing increased risks associated with radical environmental and societal changes. Broader externalities now more than ever impact airline operations and consequently its ability to deliver experiences to passengers the way they were designed (Verhoef et al. 2009). Extreme weather (e.g., Hurricane Dorian) is being experienced worldwide as a result of climate change, and outbreaks such as the Covid-19 pandemic are creating more frequent disruptions in airline operations. Technological challenges have also created significant disruptions to the industry (e.g., the grounding of the Boeing 737 Max), as has political (e.g., protests in Hong Kong) and geopolitical (e.g., Venezuelan embargo, the war in Ukraine) unrest. While these factors are external to the industry, mergers and acquisitions continue in most regions as well, thus creating further oligopolistic competition. Importantly, due to the instability and inequality of the Latin American market, major Latin American airline companies may be facing the most significant risks on the continent. As a result, repeat business and customer loyalty should become more important than ever under these circumstances.

Nevertheless, how can these two goals be achieved by airlines in an increasingly competitive and uncertain environment? While customer loyalty programs may be helpful tools in this regard, they could prove to be a 'double-edged sword' due to increased customer expectations and entitlements (Ma et al. 2018). We argue that as a consequence of the current state of the airline industry, brand trust has become an increasingly important deciding factor for airline passengers. Verhoef et al. (2002) found that when it comes to providing services, trust is linked to positive referrals from customers who have already used a service and liked it, so they started telling other people about it. According to the authors, customers recommend a service because they trust it. This is important for the development of a good relationship between the customer and the service provider. Song et al. (2019) show that, particularly in the airline industry, the perceived quality of service has an essential effect on how the customer perceives the service. They argue that trust in the service provider grows when a customer experiences high quality. Han and Hwang (2015) say that trust helps solidify and promote a long-term relationship between the service provider and the service user, therefore improving the performance efficiency of resources used for marketing, communication, and research, which are all needed to acquire and keep customers. However, it is important to note that the trust placed on a brand is the product of a complex customer's assessment of both internal and external touchpoints that make up the customer experience provided by the airline. Service is a critical component of customer experience, and Hu et al. (2009) argue that high-quality services, in particular, can lead to a greater sense of value and customer satisfaction. This directly affects the company's brand image and increases customer loyalty. When addressing service experience, in particular, Bezerra and Gomes (2019) say that passengers' expectations and satisfaction are connected to a good brand image. Song et al. (2019) further highlighted the importance of the brand by arguing that a company's corporate image is tied not only to customer retention but also to customer perception of the company as a whole and whether or not the appreciation of the experience received was strong enough to use the same airline again.

Therefore, holding brand trust as a critical element of the creation of customer loyalty for service industries, the purpose of this research is to examine the drivers of brand trust in a Latin American airline. In order to accomplish this goal, an augmented version of the AIRQUAL model was proposed, along with the introduction of a measure of CX. The model evaluated both the direct impact of the dimensions of AIRQUAL on brand trust, how CX impacted brand trust directly, and how it mediated the relationship between the dimensions of AIRQUAL and brand trust. The study was conducted with passengers of Avianca, a well-known and respected brand in Latin America. CX was assessed using a measure proposed by Kim and Choi (2013) and Brady and Cronin (2001). AIRQUAL is a model proposed by Nadiri et al. (2008) for assessing the quality of airline service consisting of five dimensions: airline tangibles, terminal tangibles, personnel services, empathy, and image. The original dimensions were expanded with two additional touchpoints identified in the literature to represent both internal and external touchpoints that make up the customer experience resulting in a more robust research model. The additional dimensions, namely, the impact of perceptions associated with other customers and the process of the purchase experience, were incorporated to account for a more holistic assessment of the provided experience and account for the three stages of the quality of airline service proposed by Namukasa (2013): before the flight, during the flight, and after the flight. The results of this study suggest that all three areas—pre-flight service quality, in-flight service quality, and post-flight service quality—are equally important and significantly impact customer perception of quality.

## Literature review

### Customer experience (CX)

CX is a multidimensional notion that results from a customer's brand-related stimuli response (Lemon and Verhoef 2016) and is hypothesized to have cognitive, emotional, behavioral, sensory, and social dimensions (Verhulst et al. 2020), meaning that the focal experience object determines CX's dimensions, ranging from macro to micro level objects (Vargo and Lusch 2017). The challenge associated with successfully implementing an experience is that it is a concept defined so broadly that hardly anything is excluded, and anything that explains customer dissatisfaction or delight is considered part of an experience. It has been argued that the experience should be built from the bottom up, focusing on individual elements rather than holistically, allowing for the development of a systematic and sustainable change program (Maklan et al. 2017). Some studies argue that CX can be developed and delivered to customers (Hamilton and Wagner 2014). In contrast, others posit that CX emerges in customers' lives and cannot be managed directly (Helkkula and Kelleher 2010). In a more reconciling view, Becker and Jaakkola (2020) propose that the two research traditions can be bridged by relying on their common denominator that CX results from responses to varied stimuli. As corporations cannot directly control consumer responses, they cannot control CX in its totality. That leaves them the option to control customer stimuli to attempt to influence consumer responses.

Voorhees et al. (2017) underlined that CX occurs across several interactions pertinent to a core service offering, including multiple "moments of truth" in the form of touchpoints that impact customer outcomes. Touchpoints represent individual connection events between the brand/firm and customers during the customer journey, such as information gathering, payment, delivery, and consumption (Homburg et al. 2017). Touchpoints can be analyzed in terms of control, nature, and customer journey stage. Touchpoint control identifies who controls customer–brand interactions. Store environment, layout, advertising, and employees are some of the touchpoints that can be controlled by the organization, whereas customer behavior, influencers, or other brands or firms are typically considered uncontrollable touchpoints (Kranzbühler et al. 2019). While the CX literature is predominantly focused on firm-controlled touchpoints, non-firm-controlled touchpoints are increasingly attracting the attention of researchers, particularly the role of other customers (Gao et al. 2019; Siqueira et al. 2019). CX researchers acknowledge the uncontrollable trait of CX by a single brand/firm, supporting the notion of the complexity of customer journeys and the various actors that can impact it. However, even though some actors cannot be fully controlled, in the case of customers, they can be influenced by the experience provided to take up active roles as brand ambassadors, influencers, or promoters through offline and online Word Of Mouth (WOM) (De Keyser et al. 2019; Siqueira et al. 2019). Touchpoint nature reflects brand/firm representation. According to De Keyser et al. (2019), the nature of touchpoints can be human, digital, physical, or a combination. These authors argue that the touchpoint journey stage represents the location of touchpoints along the customer journey and can be identified as pre-purchase, purchase, and post-purchase. Pre-purchase refers to processes (need recognition or information search, for example) that occur before the purchase decision (Puccinelli et al. 2009). The purchase stage represents the intermediary point of the customer journey where purchase decisions and actions occur. Lastly, post-purchase touchpoints represent the aftermath of the purchase and usually refer to product usage, brand communities (Carù and Cova 2015), or product-return points (Lemon and Verhoef 2016).

### Brand trust

Brand trust, considered the core of brand value (Kotler et al. 2010), is a concept strongly connected to expectations about brand reliability (Munuera-Aleman et al. 2003) and, moreover, generates brand loyalty (Atulkar 2020; Chaudhuri and Holbrook 2001; Laroche et al. 2012). Brand trust involves all the knowledge and experience—favorable or not—about a brand. Accordingly, firms need to develop strong relationships with consumers (in particular, Chaudhuri and Holbrook 2001 highlight that trust is related to consumers’ willingness to rely on the capacity of the brand to perform its function) from a perspective of loyalty and brand equity (Husain et al. 2022) also because it positively contributes to brand performance, given its influence on brand commitment (Delgado‐Ballester and Luis Munuera‐Alemán 2001). Lantieri and Chiagouris (2009) studied how a mistrust climate can affect brand trust, revealing eight themes that generate mistrust: the emergence of more cynical consumers, frequent consumer recalls, emphasis on the needs of business owners over the needs of consumers, company structure, uneven quality advances, service quality declines, too many undifferentiated choices, and pseudo relationships.

The role of brand trust in airline services has attracted academic attention lately. The role of trust is deepened, in fact, by Erkmen and Hancer (2015) by analyzing 523 flight attendants of an airline company. Specifically, they found that brand trust positively influences the brand commitment of employees. Moreover, brand trust is positively connected with the brand citizenship behaviors of employees (Kim et al. 2018). Jamuary et al. (2015) studied, in Malaysia, those factors which contribute to airline brand trust, finding that WOM, security/privacy, perceived risk, good online experience/brand reputation, and perceived quality of information contribute to brand trust for airline services in online environments (Ha 2004). Additionally, Lin and Ryan (2016) studied the role of branding, which involved 518 passengers in Taiwan. They found a positive association between airlines' mission statements and passengers' perceptions of brand trust. In particular, they noticed that brand trust acts as a mediator between brand equity and passengers' perception. Particularly relevant is also the relationship between global airline alliances and brand strategies. From this point of view, Wang (2014) explored the influence of alliances on purchase intentions in Taiwan, showing that there is a positive relationship between global alliances, brand equity, brand preferences, and purchase intentions and that there is a significant impact of alliances on brand equity and brand preferences on purchase intentions in case of highly involved passengers. Han et al. (2019) focused on airline passengers' repurchase decision-making process when investigating purchase intention. They found that in-flight core-product and service-encounter quality, brand attitude, brand image, brand trust, and brand love positively affect repurchase intention. Han et al. (2019) also identified a significant difference between low-cost and full-service airlines in brand attitude, brand image, brand trust, and brand love. According to Koklic et al. (2017), service staff quality, more present in full-service airlines, is more essential than tangibles when assessing passengers' happiness with airline services. Law et al. (2022) studied Laos passengers and found a positive association between repurchase intention, service quality (influenced by brand credibility, product originality, and loyalty programs), and customer happiness. Service quality and trust are also critical factors for airline brand equity as they drive repurchase, according to Saleem et al. (2017). Law et al. (2022) recently emphasized the need to focus on brand equity, starting with product differentiation to impress consumers and increase repurchasing. While all brand components affect the sense of value, and brand image affects service quality and company image, brand trust impacts service quality more than consumer value. Sirdeshmukh et al. (2002) link the latter to airline loyalty.

### AirQual model

The first tool to measure the quality perception of airline passengers was proposed by Parasuraman et al. (1985), whose model, named SERVQUAL, consisted of specific dimensions, such as tangibles, reliability, responsiveness, assurance, and empathy. However, as also explored by (Robledo 2001), this model is not necessarily the most appropriate scale to assess service quality. In fact, with the aim to measure service quality rather than expectations (Ali et al. 2021), SERVPERF was introduced (Rodrigues et al. 2011), which was proposed by Cronin and Taylor (1992). The latter model, however, was criticized for being too general (Shen and Yahya 2021). Once the limits of the past models were ascertained, Ekiz et al. (2006) proposed a new one to measure airline service quality, named AIRQUAL, consisting of five dimensions, namely airline tangibles, terminal tangibles, personnel services, empathy, and, finally, image (Farooq et al. 2018). This model was validated by Nadiri et al. (2008). Below, Table 1 briefly compares the three models’ dimensions (Hasan et al. 2019).

**Table 1:** Comparison of SERVQUAL, SEVRPREF, and AIRQUAL models across their corresponding dimensions

Model	Dimensions
SERVQUAL (Parasuraman et al. 1988)	Tangibles, reliability, responsiveness, assurance, empathy
SERVPERF (Cronin and Taylor 1992)	Reliability, assurance, tangibility, empathy, and responsiveness
AIRQUAL (Ekiz et al. 2006)	Airline tangibles, terminal tangibles, personnel, empathy, and image

The aforementioned five dimensions of the AIRQUAL model will be further explored in the following paragraphs

#### Airline tangibles

Airline tangibles are cues connected with service quality, including interior and exterior types of equipment, catering services, seats, and the cleanliness of toilets and air conditioning (Shen and Yahya 2021; Suki 2014). Research showed that these are among the most critical elements to consider in terms of quality. Alsini (2017) reported them as the most influential factor in generating satisfaction in airline customers. Nadiri et al. (2008), for instance, found that tangibles not only influence customer satisfaction but also affect repurchase intention. Similarly, Kim and Lee (2011) observed that tangibles and responsiveness are the most relevant predictors of customer satisfaction and retention for low-cost carriers. In contrast, note that Suki (2014) argues that airline tangibles do not significantly impact customer satisfaction.

#### Terminal tangibles

Terminal tangibles include those services more connected to the airport sign boards, security, control system, air conditioning on the terminal, shops, parking services, the size of the airport, effectiveness of signage, availability of trolleys, information counters, and the comfort level of waiting in the hall of the airport (Abdel Rady 2018; Farooq et al. 2018). Terminal tangibles are one of the most visible dimensions (Farooq et al. 2018). According to Ekiz et al. (2006), terminal tangibles positively affect perceived service quality. Similar to airline tangibles, research has shown that terminal tangibles influence customer satisfaction (Wattanacharoensil et al. 2016) (Wattanacharoensil et al. 2016). According to Suki (2014), however, parking spaces, airport capacity, number of shops, availability of trolleys, conformable waiting halls, air conditioning, and reliable security controls may not be so relevant when assessing the influence on customer satisfaction.

#### Personnel services

This dimension is strictly connected to airline staff and flight attendants (Farooq et al. 2018). The role of personnel services is fundamental for passenger satisfaction. Babbar and Koufteros (2008) highlight that higher satisfaction levels require individual attention, helpfulness, courtesy, and promptness. The latter means points to the necessity of paying attention to human resources management to motivate employees to facilitate their customer approach. This has also been placed in evidence by Ringle et al. (2011), who found that perceived safety influences travelers' perceived pleasure, with positive consequences on customer loyalty. Recently, regarding low-cost and full-service airlines, the influence of personnel quality on satisfaction has also been confirmed by Koklic et al. (2017), particularly concerning customer satisfaction and behavioral intentions.

#### Empathy

Empathy in this study's context relates to luggage handling, ticketing services, and compensations in case of loss. Empathy is recognized as an indicator of quality in business communication (Fuller et al. 2021), which triggers customer loyalty and repeat buying behaviors (Bahadur et al. 2018; Chang and Yeh 2002). On the other hand, the role of empathy has also been acknowledged in the literature as twofold. In particular, Wieseke et al. (2012) saw that customer satisfaction is strengthened not only by employee empathy but also by the customers. Meanwhile, Moslehpour et al. (2018), Humphrey (2013), and Cardon et al. (2012) highlighted how empathy and emotional intelligence benefit general entrepreneurship. Interestingly, An and Noh (2009) studied the differences between customer seat classes, revealing that empathy is a predictor of quality, both in the prestige class (along with alcoholic and non-alcoholic beverages, responsiveness, reliability, assurance, the presentation style of food, and food quality), as well as in the economy one (along with responsiveness, food quality, alcoholic beverage, non-alcoholic beverage, and reliability).

#### Image

As in any business, image plays a crucial role. Hence, developing a good brand reputation is fundamental to improving customer satisfaction (Shen and Yahya 2021). Image is, in fact, one of the most critical factors in evaluating a company and understanding customers’ choices (Fombrun 1995). Farooq et al. (2018) argued that it might be necessary to associate airline image with frequent flyer programs and promotional offers to maintain a good image. Ekiz et al. (2006) also showed that image affects the customers’ perceived service quality, involving value for money, promotional offers, and goodwill. The relevance of corporate image has also been reported by Song et al. (2019). Their study revealed that responsiveness, and reliability, had a significant effect on corporate image and customer trust, and, in addition, it encourages a positive assessment of the enterprise by the customers.

### Purchase experience

Purchase decision-making is a process that involves different choices by consumers, and where different psychological processes are involved when it occurs (e.g., which kinds of needs does the purchase satisfy? Are there social factors that influence consumer choices?). Kotler et al. (2009) succinctly explained the whole process across five main stages: (1) problem recognition; (2) information search; (3) evaluation of alternatives; (4) purchase decision; and (5) post-purchase behavior.

Indeed, the role of the purchase decision process in the airline CX context is of great interest. For instance, Tarkang et al. (2022) evaluated Turkish Airlines consumers' purchasing intentions using electronic WOM (i.e., free advertising that reinvigorates a brand and boosts sales by increasing purchase rates; Kietzmann and Canhoto 2013), where airline tickets are increasingly being purchased online (Gupta et al. 2004). The main findings relate to having a user-friendly and attractive website as critical to repeat internet purchases and referrals. Habit, price savings, performance expectancy, and facilitating factors also influence internet purchases (Escobar-Rodrguez and Carvajal-Trujillo 2013). The cultural environment has also been found to affect internet shopping in European customers (Ruiz-Mafe et al. 2013).

A brand's strategic role in decision-making is also crucial (Jara and Cliquet 2012; Kumar and Hansted Blomqvist 2004; Smith and Brynjolfsson 2001), and in the aviation industry, this is no exception (Cervera-Taulet et al. 2013; Dirsehan and Kurtuluş 2018; Hwang and Hyun 2017). For instance, airline brand credibility increases purchase intention by facilitating decision-making and boosting emotional commitment (Jeng 2016). A favorable association between global airline alliances, brand equity, and brand choice for highly involved travelers can increase purchase intention (Wang 2014). Price perception can also differ between LCC and TA, where the price tends to be the most significant factor only for LCC passengers (Mikulić and Prebezac 2011).

### Other customer perception

Global changes are constantly affecting the airline business (Nelms 1991) along with the consumer perceptions of airlines. Passengers have higher expectations and are becoming more sophisticated (Kaynak et al. 1994). The views of other customers can also affect service enjoyment and expectations (Brocato et al. 2012; Klaus and Maklan 2013; Siqueira et al. 2019). Studying the role of airline travelers’ interactions has actually led to the development of a passenger anger scale (Ye et al. 2022). Brochado et al. (2019), for instance, explored how internet airline reviews are linked to money ratings. Here, the conduct of customers seated together during a flight was found to affect airline experience reviews. In fact, interactions constitute the center of a consumer's social experience (Bolton et al. 2018), where active customer service is a hallmark of air travel. In general, the presence of others can affect an individual in specific ways (Lerner and Tetlock 1999). Therefore, a customer's total experience should include all touchpoints, regardless of service provider control (Palmer 2010). In this sense, we propose that passenger engagement may satisfy Lemon and Verhoef's (2016) criterion of a social, external, and independent touchpoint, prompting a favorable connection with CX. Other consumer views can further influence passenger behavior since emotions affect personal pleasure (Xu et al. 2018), whereas customer-to-customer interactions affect passenger behavior (Martin 1996). In brief, appropriate interpersonal attitudes can promote pleasantness, while violent and filthy acts do not.

Demographics may also play a distinctive role in the airline CX. Kaynak et al. (1994) found that younger passengers valued in-flight entertainment, flight attendant service, meal quality, alcoholic beverages, and frequent flyer programs. 20–30-year-olds prefer on-time flights, luggage handling, and cheap pricing. Aghabayk et al. (2021) recently noted that passenger behavior and biosecurity precautions might be more important than before as travelers are more worried, fearful, and susceptible owing to the epidemic (Lamb et al. 2021). Hence, well-informed passengers will tend to trust their flight experience.

### Theoretical model

The proposed theoretical model was based on the use of the AIRQUAL model augmented with a CX metric. AIRQUAL was enhanced with two service literature dimensions: other customer perceptions and the purchase experience. The inclusion of the CX measure was based on the work previously conducted by Kim and Choi (2013) and Siqueira et al. (2019). It is also noteworthy to highlight the fact that although CS/CX/BX seem similar in concept, the literature distinguishes between them, as presented in Table 2. Therefore, the following hypothesis is posited based on the literature review:

**Table 2:** Summary of literature review

References	Service industry	Brand loyalty—LOY—examined
SQ affects CS/CX/BX		
Nadiri et al. (2008)	Airline	Yes
Namukasa (2013)	Airline	Yes
Jüttner et al. (2013)	Restaurant	No
Kim and Choi (2013)	General	Yes
Rajaguru (2016)	Airline	No
Priporas et al. (2017)	Airbnb	No
Farooq et al. (2018)	Airline	No
Kumar and Kumar (2019)	Airline	No
Ribeiro and Prayag (2019)	Restaurant	No
SQ affects BT		
Esmaeilpour et al. (2017)	Banking	No
Han et al. (2019)	Airline	Yes
SQ affects BT through CS/CX/BX		
Walter et al. (2010)	Restaurant	No
So et al. (2016)	Airline	Yes
Şahin et al. (2017)	Automobile	Yes
Simarmata et al. (2017)	Airline	Yes
Erkman and Hancer (2019)	Restaurant	Yes

*SQ* service quality, *CS* customer satisfaction, *CX* customer experience, *BX* brand experience, *BT* brand trust

#### H1

Customer experience mediates the relationship between the dimensions of service quality and brand trust.

## Methodology

### Sampling

The population studied were travelers based in Bogota (Colombia) who had flown with Avianca (a major Latin American airline serving most of the American continent, as well as strategic European destinations) as tourists in the past three months. Data were collected using snowball sampling; the instrument was created in Google forms and initially e-mailed to MBA students who, in turn, asked friends and colleagues to participate and share the survey with others through social media. Data were collected from July 22–25, 2019.

### Measuring instrument

Nadiri et al. (2008) validated the AIRQUAL instrument to measure service quality in the airline industry. It comprises five dimensions: airline tangibles, terminal tangibles, personnel services, empathy, and image. A modified version of this instrument was used to measure service quality (Farooq et al. 2018). In order to emphasize the purchase experience, five questions from the Dabholkar et al. (1996) instrument were added. CX was measured using the three questions from the Kim and Choi (2013) instrument and two from the Brady and Cronin (2001) instrument. Perceptions of other customers were measured using the Brocato et al. (2012) instrument. Brand trust was measured using the Chaudhuri and Holbrook (2001) instrument. Respondents were asked to rate all questions on a five-point Likert scale. Questions were presented in randomized order. The questionnaire is presented in the Appendix.

### Procedure

#### Method of analysis

The hypotheses were tested using PLS path modeling (XLSTAT version 2019.1.2). The PLS algorithm was used to estimate inner model path coefficients to avoid problems of multicollinearity resulting from OLS (Tenenhaus and Vinzi 2005).

## Results

Based on the procedure, a total of 406 usable responses were obtained. The demographic profile of respondents is presented in Table 3.

**Table 3:** Demographics profile of respondents from usable responses

*Sex*	%	*Employment*	%
Male	38.9	Unemployed	3.7
Female	61.1	Student	7.1
		Self-employed	34.0
*Age*	%	Employed	55.2
18–24	7.1		
25–34	33.3	*Income*	%
35–54	30.3	Up to COP*$3 million**	24.9
55 and older	29.2	COP$3–6 million	33.0
		COP$6–11 million	22.7
*Where purchased*	%	COP$11 million or more	19.5
Airline website	58.6		
Travel search engine	14.3	*Flying Frequency*	%
Travel website	21.7	Once per year or less	11.3
Sub-total online	94.6	Twice per year	59.9
Not online	5.4	Thrice or more per year	28.8

*COP Pesos colombianos

**1 US dollar (USD) was approximately 4,480 COP in September 2022.

A principal component factor analysis of the items in the questionnaire does not yield factors supporting the AIRQUAL dimensions of service quality. Empathy and Image did not emerge as dimensions of service quality as anticipated. Only one of the Empathy questions (EMP1"Departures and arrivals are usually on time") had a significant loading in the factor analysis and loaded together with Customer Experience variables. This makes logical sense as punctuality is an important component of Customer Experience for airlines. Two of the Image questions (IMG1 "Availability of seats and promotional offers are very much appealing to me" and IMG2 "Ticket prices are worth the services I received"), both pertaining to the fairness of prices, loaded onto a new factor which relates to Value for Money (VFM). The emergence of VFM as a dimension is supported by previous empirical studies of the airline industry (Rajaguru 2016; So et al. 2014). The third Image question (IMG3 "The airline bears a good brand image") pertains to customers' perception of the airline brand and not surprisingly loads together with Customer Experience variables. In-Flight Quality (IFQ), On-Ground Quality (OGQ), and Reservation Quality (RQ) emerged as factors. The Personnel Services did not emerge as a factor, but the questions from AIRQUAL split across IFQ, OGQ, and RQ, providing a human element to each of these constructs. Details of the factor structure for the sample are presented in Table 4. It is interesting to note that questions EMP1 (punctuality) and IMG3 (brand image) load onto customer experience (CX). Other customer perceptions (OCP) and brand trust (BT) factored as anticipated.

**Table 4:** Evaluation of measurement model

Construct	Item	Loading	CR	AVE
In-flight quality (IFQ)	AT1	0.702	0.923	0.600
	AT2	0.792		
	AT3	0.715		
	AT4	0.793		
	AT5	0.797		
	AT6	0.785		
	PS5	0.790		
	PS6	0.815		
On-ground quality (OGQ)	TT2	0.721	0.915	0.604
	TT3	0.697		
	TT4	0.801		
	TT5	0.809		
	PS1	0789		
	PS2	0.816		
	PS4	0.813		
Reservation quality (RQ)	PS3	0.799	0.865	0.671
	PX4	0.842		
	PX5	0.835		
Value for money (VFM)	IMG1	0.874	0.867	0.757
	IMG2	0.874		
Other customer perception (OCP)	OC1 OC2 OC3 OC4	0.839 0.889 0.939 0.937	0.946	0.813
Customer experience (CX)	EMP1	0.775	0.952	0.738
	IMG3	0.813		
	CX1	0.913		
	CX2	0.849		
	CX3	0.895		
	CX4	0.907		
	CX5	0.852		
Brand trust (BT)	BT1	0.939	0.946	0.815
	BT2	0.954		
	BT3	0.879		
	BT4	0.833		

*CR* composite reliability, *AVE* average variance extracted

The Harman test indicates that 43.0% of covariance is accounted for by a single factor, indicating that any common method variance present is unlikely to create any bias (Fuller et al. 2016).

The absolute and relative goodness of fit indices are 0.623 and 0.940, respectively, and both are suggestive of a good model. Composite reliability for all constructs in the model exceeds 0.85 (refer to Table 4), indicating internal reliability. All factors have an AVE greater than 0.5, and all but one of the variable loadings exceeded 0.7 (0.697 reported, refer to Table 4), indicating convergent validity. Discriminant validity is illustrated in Table 5.

**Table 5:** Discriminant validity

	IFQ	OGQ	RQ	VFM	OCP	CX	BT	AVE
IFQ	1							0.600
OGQ	0.454	1						0.604
RQ	0.229	0.399	1					0.671
VFM	0.139	0.153	0.120	1				0.757
OCP	0.378	0.279	0.170	0.101	1			0.813
CX	0.351	0.253	0.190	0.329	0.337	1		0.738
BT	0.285	0.226	0.203	0.197	0.218	0.552	1	0.815
AVE	0.600	0.604	0.671	0.757	0.813	0.738	0.815	

Neither OGQ nor RQ significantly influences either CX or BT. In the case of OGQ, this is probably because most of the questions relate to the airport itself rather than the airline. In the case of RQ, 41.4% of the sample did not make their reservation using the airline website and thus interacted with other parties. OCP has a significant influence on CX but none on BT. Because CX has a significant influence on BT, implying that the relationship between OCP and BT is fully mediated by CX. IFQ and VFM have a significant effect on both CX and BT, and CX has a strong effect on BT. This implies that CX partially mediates the relationship between these two dimensions of service quality and BT. As IFQ and VFM are the only dimensions of service relevant to the brand, H_1_ is thus supported.

Squared correlations are reported in the matrix. For all constructs, AVE > squared correlations with other constructs implying discriminant validity.

The path coefficients for the model are presented in Table 6.

**Table 6:** Path coefficients

Path	Path coefficient
IFQ → CX	0.210***
OGQ → CX	0.045
RQ → CX	0.067
VFM → CX	0.376***
OCP → CX	0.279***
IFQ → BT	0.092**
OGQ → BT	− 0.017
RQ → BT	0.089
VFM → BT	0.118*
OCP → BT	0.050
CX → BT	0.551**

***, **, *Significant at the 1%, 5%, and 10% levels, respectively.

Based on the path coefficients presented in Table 6, Hypothesis 1 is partially supported. CX partially mediates the relationship between in-flight quality, value for money, and brand trust. On-ground quality and reservation quality have no significant relationship to either CX or brand trust. CX fully mediates the relationship between other customer perceptions and brand trust.

## Discussion

This research aimed to examine the drivers of brand trust in the Latin American airline industry. An augmented model of AIRQUAL with the inclusion of a measure of CX was used to assess the quality perception of the airline service of Avianca and its impact on brand trust. AIRQUAL was updated with two dimensions identified in the service literature: the impact of perceptions associated with other customers and the process of the purchase experience. The dimensions of the updated AIRQUAL model were used to evaluate CX as previously done by Kim and Choi (2013) and Siqueira et al. (2019), where CX is not the result of a second-order model as proposed by Klaus and Maklan (2013) but rather an objective measure of the construct. The individual impact exerted on brand trust by CX and of each of the dimensions of AIRQUAL produced in a CFA was analyzed. The results presented in this article provide a rare glimpse of service encounters in a developing country in Latin America and address the emerging question of how Latin American airlines can achieve repeat business and customer loyalty in increasingly competitive and uncertain environments. The results suggest that specific key drivers of airline perceived service quality in the augmented AIRQUAL model will significantly affect CX, which mediates the relationship with brand trust.

The inclusion of the CX construct in the AIRQUAL model yielded promising results. Beyond examining service quality exclusively in the air travel industry, CX helps provide a more holistic evaluation of the experience lived by customers with legacy dimensions of the AIRQUAL model and the inclusion of the dimensions of other customers and the process of the purchase experience. The principal component factor analysis of the items in the AIRQUAL instrument did not yield factors supporting the AIRQUAL dimensions of service quality. The resulting dimensions still produced a robust model that can effectively assess the air travel CX. It captures the essential aspect of the three categories into which services are commonly divided: before the flight (when procedures such as flight booking and check-in procedure occur) as represented by (RQ) and (OGQ), during the flight (when buyers get to experience airplane features such as seat comfort and cabin cleanliness) represented by (IFQ), and after the flight (when dealing with landing procedures and luggage delivery) represented by terminal services as well as (VFM) (Bellizzi et al. 2020).

The impact of human interactions during an experience was also captured through the personnel service questions from AIRQUAL split across IFQ, OGQ, and RQ. In contrast, online interaction was captured by the split between online versus in-person ticket purchases. Even though neither OGQ nor RQ was found to influence either CX or BT significantly, it is important to assess air travel holistically by examining services provided by the airline and the ensuing association with services provided by air terminals to passengers. In the case of RQ, 41.4% of the sample opted to bypass the airline website and interact with other parties instead. While IFQ and VFM had a significant effect on both CX and BT, it is important to highlight the strength of the effect CX has on BT. The implication that CX partially mediates the relationship between these two dimensions of service quality and BT is profound and shows remarkable promise for the inclusion of CX in the evaluation of airline experience.

The mediation of the relationship between OCP and BT by CX was another relevant result that further supported the proposed research model. The addition of CX to the AIRQUAL model helps capture different touchpoints not previously captured that can better explain how CX can help engender BT. This is important because brand trust can be influenced by different variables (Portal et al. 2019); capturing different touchpoints through CX and the service dimensions examined becomes extremely valuable.

## Theoretical implications

According to the marketing literature, improved service quality is widely accepted as a critical success factor in this day of intense competition (Tsoukatos and Mastrojianni 2010). Due to the nature of services, service quality evaluation has been the topic of countless studies, and due to its conceptual and empirical link to customer satisfaction, service quality has become a central marketing strategy. This study shows that the use of AIRQUAL when measuring service quality relative to customers' experience of the brand or their trust in the brand may be challenging. Customers generally experience many touchpoints when undertaking a flight, which creates their total experience of the event. However, not all of these relate to their experience of the airline brand itself (e.g., making a reservation using a travel agent or travel website, conditions at and convenience of the airport, airport security, and baggage handling). Hence, it becomes essential to distinguish between the overall experience to the more specific brand experience. This makes the inclusion of CX and additional dimensions to the AIRQUAL model a valuable contribution to the Air travel marketing literature.

## Practical implications

It is essential to highlight that even though the overall travel experience and the experience of the airline brand differ, they remain closely connected as complementary services that should be examined as a holistic experience. This research demonstrates that the key drivers of airline CX and brand trust are

- the physical aspects of the flight;
- interaction with the flight crew;
- perception of the flight representing good value for money.

Furthermore, the perception of the behavioral aspects of other passengers affects customers' experience and, indirectly, brand trust.

These variables can contribute to the improvement of the experience provided by airlines in Latin America. This is important because a high-quality experience perception can impact customers' value assessment when evaluating money, time, and energy expenditure relative to the quality of service received and leading to the retention of high perceived value service providers and high satisfaction with the experience (Hapsari et al. 2016; Howat and Assaker 2013). Another obstacle to offering high-quality services is failing to grasp clients' genuine needs and desires (Izogo and Ogba 2015). The current competitive market environment has prompted airlines to prioritize cost reduction in order to achieve effective company operations, yet doing so frequently comes at the expense of service quality and customer pleasure (Boetsch et al. 2011). The proposed model provides a unique map that can help managers direct their efforts to areas that can most significantly impact the experience provided to their customers.

A brand represents one of the most valuable intangible assets that firms own (Keller and Lehmann 2006), capable of influencing consumers' choices (Degeratu et al. 2000; Yeung and Wyer 2005) as it is a critical element of marketing strategy (Zeren and Kara 2021). The role of the brand is, moreover, relevant to developing long-lasting relationships with consumers in particular. Tuškej et al. (2013) found that they can also contribute to building consumers' identity, especially in the case of congruity between consumers and brand values, with implications on consumer behavior. With regard to the impact of brand trust on future purchases, Esch et al. (2006) highlight that brand knowledge does not affect them. However, it is necessary in order to build a strong relationship between consumers and a familiar brand with a positive image. In addition, this study also revealed that brand satisfaction and brand trust are critical antecedents of current and future purchases. Brand trust is also connected to brand reputation, which is, in turn, linked to brand reliability (Afzal et al. 2010) and, consequently, to the possibility of repeating a purchase. For instance, Han et al. (2015) studied this relationship in the food sector, revealing that food and service quality, brand affect, brand awareness, and brand association positively influence brand trust, while self-congruence is actually negatively connected to trust.

In this region of radical environmental, social, and technological challenges, airline management must ensure that the actual flight experience is safe, comfortable, and pleasant for all passengers. When a disruption occurs, how the airline deals with the aforementioned key drivers will most likely be determinant concerning the perceptions of the experience and trust of the brand.

## Limitations and future research

First, using an online survey to collect data with the instruction that participants should respond based on a recent airline experience meant that some participants might not be able to produce accurate evaluations of the airline experience after using them. This issue was discussed by Cutler et al. (1996), who noted that people's memories fade when they talk about the past. Second, this study had a relatively small sample size compared to the target group, air travelers in Latin America. In order to obtain a more representative sample, methods other than convenience sampling should be utilized. Additional research with other companies in the same industry should be done to make comparisons and gain additional insights.

In terms of future areas of study, the impact of self-service technologies offers a promising avenue to help expand the understanding of the impact of technology on the airline travel experience. The ramifications that result from the characteristics of self-service technologies require further study. The market now revolves entirely around technology. Customers are being offered the opportunity to employ self-service technologies, altering how they connect with businesses to improve service results and give them more leisure time while flying (Ku and Chen 2013; Meuter et al. 2000). Airports keep implementing cutting-edge self-service technologies, like mobile check-in, self-check-in kiosks, baggage drop, security, and immigration inspections. Self-increasing service significance is fundamentally altering the way in which services are provided at airports. In order to properly comprehend the context of airline CX, future studies should take into account the purpose of the journey, the traveler's income, their country, the ownership of the airport, and the level of regulation.

## Appendix A

**Table:** 

Construct/ Question	Reference	Code
*AIRQUAL*		
Airline Tangibles		
Aircraft was equipped with latest and modern technology Quality of catering service was good Quality of air conditioning in the planes was good Interior of aircraft was well maintained Seats were clean and comfortable Cleanliness of the plane toilets was well maintained	(Farooq et al. 2018)	AT1 AT2 AT3 AT4 AT5 AT6
Personnel Services		
Airline staff was well dressed Workers were well aware of their duties Ticketing and reservation service was error free Whenever I required airline personnel answered my questions Flight attendants were providing equal personal care to everyone Workers were willing to extend their help to everyone on plane	(Farooq et al. 2018)	PS1 PS2 PS3 PS4 PS5 PS6
*Terminal tangibles*		
Quality of air conditioning on airport terminals Information counter was readily available to assist me Number of shops were adequate for my needs Airport had effective sign boards Security and control system were friendly and reliable Adequate number of trolleys were available on airport Cleanliness of the airport toilets was well maintained	(Farooq et al. 2018)	TT1 TT2 TT3 TT4 TT5 TT6 TT7
*Image*		
Availability of seats and promotional offers are very much appealing to me Ticket prices are worth the services I received The airline bears a good brand image	(Farooq et al. 2018)	IMG1 IMG2 IMG3
Purchase Experience		
Airline makes it easier for customers to find what they need Airline provides its services at the time it promises to do so Airline has what customers need available when the customers want it Customers feel safe in their transactions with the airline Airline accepts all major credit cards Airline makes it easier for customers to find what they need	(Dabholkar et al. 1996)	PX1 PX2 PX3 PX4 PX5 PX6
*Other Customer Perception*		
The behavior of the other customers was appropriate for the setting Other passengers were friendly toward me I found that the other passengers behaved well Other passengers’’ behavior was pleasant	(Brocato et al. 2012)	OC1 OC2 OC3 OC4
*Customer Experience*		
I would say that the experience with the airline is excellent I believe that we get superior experience with this airline I think that total experience procedure with this airline is excellent When I leave this airline, I usually feel that I had a good experience I believe this airline knows the type of experience its customers want	(Brady and Cronin 2001) (Lemke et al. 2011)	CX1 CX2 CX3 CX4 CX5
*Brand Trust*		
I trust this airline brand This airline brand is reliable This is an honest airline brand This airline brand is safe	(Chaudhuri and Holbrook 2001)	BT1 BT2 BT3 BT4

## Data Availability

Data will be made available upon request.
